# Production of H_2_-Rich Syngas From Lignocellulosic Biomass Using Microwave-Assisted Pyrolysis Coupled With Activated Carbon Enabled Reforming

**DOI:** 10.3389/fchem.2020.00003

**Published:** 2020-01-24

**Authors:** Kaiqi Shi, Jiefeng Yan, J. Angel Menéndez, Xiang Luo, Gang Yang, Yipei Chen, Edward Lester, Tao Wu

**Affiliations:** ^1^Key Laboratory for Carbonaceous Wastes Processing and Process Intensification Research of Zhejiang Province, The University of Nottingham Ningbo China, Ningbo, China; ^2^College of Science & Technology, Ningbo University, Ningbo, China; ^3^Instituto Nacional del Carbón, CSIC, Oviedo, Spain; ^4^Department of Chemical and Environmental Engineering, The University of Nottingham, Nottingham, United Kingdom

**Keywords:** biomass, microwave-assisted pyrolysis, reforming, activated carbon, hydrogen, syngas

## Abstract

This study focuses on the use of a microwave reactor that combines biomass pyrolysis, at mild temperature, with catalytic reforming of the pyrolytic gas, using activated carbon, for generating hydrogen-rich synthesis gas. The traditional pyrolysis of biomass coupled with the reforming of its pyrolytic yields were also conducted using an electrically heated reactor. The bio-oil attained from conventional pyrolysis was higher in comparison to the yield from microwave pyrolysis. The reforming of the pyrolytic gas fraction led to reductions in bio-oil yield to <3.0 wt%, with a simultaneous increase in gaseous yields. An increase in the syngas and H_2_ selectivity was discovered with the reforming process such that the use of microwave pyrolysis with activated carbon reforming produced 85 vol% synthesis gas fraction containing 55 vol% H_2_ in comparison to the 74 vol% syngas fraction with 30 vol% H_2_ obtained without the reforming. Cracking reactions were improved with microwave heating, while deoxidation and dehydrogenation reactions were enhanced by activated carbon, which creates a reduction environment. Consequently, these reactions generated H_2_-rich syngas formation. The approach implemented in this study revealed higher H_2_, syngas yield and that the overall LHV of products has huge potential in the transformation of biomass into high-value synthesis gas.

## Introduction

The escalating concerns over energy supply security and deterring environmental implications related with fossil fuels consumption have made searching for sustainable and alternative energy resources very attractive in the past few decades (Mushtaq et al., 2014; Zhao X. et al., 2017). The contribution prospects of biomass as an alternative energy resource remains substantial in the low carbon economy future (Baliban et al., 2013; Motasemi and Afzal, 2013). As a result, the investigation of effective, non-polluting and affordable methods for the large-scale utilization of biomass is crucial.

Thermochemical technologies are still the main approach for generating high value chemicals or energy using biomass as the feedstock (Luque et al., 2012; Sikarwar et al., 2016), which includes pyrolysis, combustion, gasification, and catalytic reforming process etc. In the past, biomass pyrolysis has been comprehensively explored and applied to produce gaseous fraction, bio-oil and bio-char from numerous feedstocks (Kirtay, 2011; Melero et al., 2012). The use of microwave irradiation to enhance and accelerate pyrolysis reactions is a promising approach, due to its high energy efficiency in comparison to conventional pyrolysis (Kirtay, 2011). It is reported that compared with conventional pyrolysis processes, higher fraction of gaseous yield with high syngas (CO+H_2_) and increase in quality of bio-oil was detected in microwave heating (Luque et al., 2012; Motasemi and Afzal, 2013). However, there have been only limited research done on the upgrade of pyrolytic yields from microwave assisted reactions using appropriate reforming agents (Yin, 2012; Motasemi and Afzal, 2013; Lin et al., 2014). In addition to this, previous observations from microwave processing of waste oil confirmed the gaseous and liquid products were relatively contaminant free and the char served as a metal store for efficient metal recovery (Lam et al., 2016a). Apart from the quantity, the quality of the yields was higher in comparison to products from conventional pyrolysis of same waste oils. Microwave processing offers benefits such as faster and more selective heating within a shorter time frame and produces more environmentally friendly products. As a result, there is an increase in research interests pertaining to the application of microwave reactors with additives for improving quantity and quality of gas and/or liquid fraction. However, the high system costs, dielectric properties of samples and the limitation of information on reactor parameters and design optimization remainsthe limitation for the widely industrial application of microwave technology (Wu et al., 2014).

Metallic catalysts, activated carbon and pyrolytic char as agents for upgrading of bio-oil and gas fraction from pyrolysis have been extensively studied (Wu et al., 2014). Metallic catalyst was used for microwave-assisted pyrolysis of waste engine oil which led to formation of 65–85 wt% of bio-oil and significant amounts of syngas (42 vol% of gas product) (Beneroso et al., 2014; Lin et al., 2014). Activated carbon's use as a microwave absorber and a reactant in the pyrolysis of waste palm cooking oil aided the generation of more bio-oil that contains more phenol and phenolics (Kuan et al., 2013; Yu et al., 2014). Other catalysts/reforming agents, such as, HZSM-5, MgO, CaO, SiO_2_ deposited HZSM-5 and Zn powder, have also been studied in both microwave-assisted and conventional pyrolysis to increase the yield of bio-oil and to upgrade quality of the bio-oil (Chen et al., 2008). Compared the studies on the upgrading of microwave pyrolysis bio-oil, there has been not much work on the upgrading of pyrolytic gas fractions, which consists of a significant portion of C1–C3 hydrocarbons. Simultaneous pyrolysis and reforming was only reported in the use of Ni-doped chars derived from rice husk as a catalyst led to a gas yield of 53.9 wt%, with 70.0 vol% of syngas in the gaseous fraction (Bu et al., 2012; Borges et al., 2014b; Lam et al., 2015; Zhang B. et al., 2015; Liu et al., 2016; Fan et al., 2017). Similarly, the use of NiO or CaO as a catalytic agent in the microwave-assisted pyrolysis of sugarcane bagasse slightly increased the production of H_2_ in the gaseous fraction (Bu et al., 2012). Microwave-assisted dry reforming and cracking of tar in the presence of char showed that a syngas rich gaseous fraction (80 vol%) was produced (Muley et al., 2016).

This work focused on the coupling of reforming enabled by activated carbon (REAC) with microwave-assisted pyrolysis of biomass (MAPB) to maximize the yield of H_2_-rich syngas at facile operating conditions. Conventional biomass pyrolysis and the reforming of pyrolytic products in a conventional reactor were also performed as a point of reference. Furthermore, the energy conversion efficiency of the process was investigated.

## Materials and Methodology

### Materials

A suite of biomass samples, i.e., bamboo, gumwood, pine and rosewood, were used in this study, which were native to Huzhou, Zhejiang Province, China. Prior to characterization and testing, about 1.0 kg of each sample was prepared using the standard procedures to ensure the representativeness of the samples and further milled to 212 μm (Zhang S. et al., 2015; Fan et al., 2017). Silicon carbide (≥1.0 mm) (SiC, Sino Reagent) was adopted as the microwave absorber for ensuring adequate heat generation and heating rates within the reaction cavity. The large particle size of SiC enabled easy bio-char segregation after the reaction. Due to the good stability and strength of SiC, the separated SiC could be reused for further used, which also reduce the cost for potential industrial application.

Coconut-derived activated carbon (Nanjing Jiali Carbon Co., Ltd.) was used as the reforming agent for upgrading the pyrolytic products. Activated carbon's capacity for absorbing microwave irradiation makes it a suitable reforming and deoxygenating agent. Before use in the reactor, the activated carbon was heated isothermally in an inert environment (N_2_) at 900°C for an hour to minimize its volatile and moisture content and ensure they remains <0.5 wt%, to avoid interactions between the pyrolytic yields and gaseous constituents of activated carbon.

### Biomass and Activated Carbon Characterization

#### Proximate and Ultimate Analyses

Proximate analysis was performed using a thermogravimetric Analyzer (NETZSCH STA449F3, Germany). The procedures are adopted from literature (Kuan et al., 2013; Hong et al., 2017). Ultimate analysis (CHNS/O) was carried out using an elemental analyzer (Perkin Elmer 2400, USA) adopting the approach reported (Wu et al., 2013). Oxygen (O) content was determined by difference. Approximately 1.5 mg of the samples were used for this test. The tests were repeated at least twice to ensure repeatability.

#### Intrinsic Reactivity Analysis

Intrinsic reactivity analysis was conducted following the procedure adopted by other researchers (Beneroso et al., 2016). In each test, ~5.0 mg of sample was used and heated in the TGA from 35 to 105°C and was kept isothermal for 30 min to remove the moisture. The temperature was then raised to 900°C and kept isothermal for 30 min. The heating rate for this entire procedure was 20°C/min and the sample was exposed to air at a flowrate of 20 ml/min. Thermogravimetric (TG) and derivative thermogravimetric (DTG) profiles were evaluated together with proximate analysis profile to determine combustion characteristics, such as ignition, devolatilization temperature, peak and burnout temperatures, etc. The methods for obtaining characteristic temperatures have been reported elsewhere (Avila et al., 2011). In addition, LHV of the sample was also calculated by using the exothermic peak area from the DSC curves (Hao et al., 2018).

#### Lignocelluloses Contents

Lignocellulosic composition of biomass samples was determined by adopting the acid detergent fiber, neutral detergent fiber, and acid detergent lignin methods that are described elsewhere (Shi et al., 2014).

### Pyrolysis Process

#### Microwave-Assisted Pyrolysis

**Figure 1:**
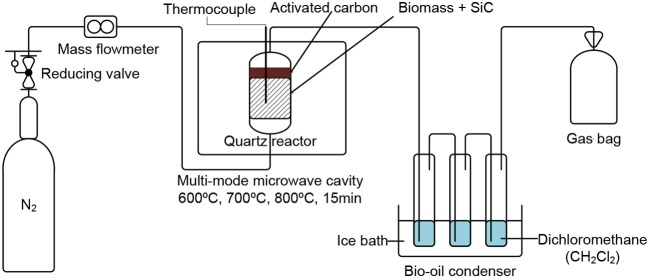
Schematic of microwave-assisted pyrolysis of biomass coupled with reforming.

Microwave-assisted pyrolysis was carried out in a multi-mode microwave-cavity (2.45 GHz, Nanjing Jiequan Microwave Co., Ltd.) with a maximum power of 3 KW. K-type thermocouple enclosed in an Niobium alloy tube was used for operating temperature detection. A customized quartz tube with similar diameter as the vertical tube furnace for conventional pyrolysis was used as the microwave reactor. Experimental set-up is illustrated in Figure 1. Approximately 5.0 g of air-dried biomass sample is used and ~5.0 g of activated carbon was placed above the SiC-biomass mix (mass ratio 5/50) in a quartz tube before being positioned in the pyrolysis reactor. The reactor was operated in temperature-controlled mode, which can be heated to 600, 700, or 800°C. The control of this temperature has been automated to avoid exceeding the set value by allowing start/stop of the microwave generator. Nitrogen (200 ml/min) was used to create an inert environment. Once pyrolysis is completed, char and SiC were separated by sieving. Ice bath was used for the condensation of bio-oil which was further dissolved in dichloromethane, while the gas yield was collected with a gasbag.

#### Conventional Pyrolysis of Biomass (CPB)

CPB was conducted in a vertical tube furnace (Tianjin Aozhan Co., Ltd.). The isothermal section of the reactor cavity was 10.0 cm long with a supporter in the middle and the inner diameter of the tube was 5.0 cm. Temperature detection was done using a K-type thermocouple. N_2_ (200 ml/min) was used to sustain an anaerobic atmosphere. In each test, ~5.0 g of air-dried sample was added into the feeder placed on top of the furnace, which was dropped into the reactor after the furnace temperature reached the pre-set temperature. This helps maintain an isothermal reaction at the desired temperature for 15 min. The tests were also carried out at 600, 700, and 800°C. The condensable volatiles were collected by using dichloromethane in an ice bath, whilst gas product was collected using gasbags.

### Characterization of Pyrolytic Products

Proximate and intrinsic analyses of char was conducted. The bio-oil was evaluated using a gas chromatograph mass spectrometry (GC-MS, Agilent 7890-5975C, USA). The GC's oven temperature was programmed to remain isothermal at 60°C for 2 min before the temperature was raised up at a heating rate of 10°C/min to 280°C and kept isothermal for 2 min. During the analysis, split mode was adopted with a split ratio of 50:1. For the correlation of the molar concentrations to the chromatographic areas, further quantification was performed using the response factors and retention time of the detected compounds. The C1–C5 hydrocarbons and other major gases of the gas yield from pyrolysis were analyzed with a gas chromatograph (GC, Shimadzu GC-2014, Japan) equipped with 2 TCD detectors, 1 FID detector, 1 HP-AL/s capillary column and 8 molecular sieve columns.

## Results and Discussion

### Properties of Raw Materials

Properties of the activated carbon and biomass are detailed in Table 1. It is evident that volatile content of the biomass is >70 wt% and fixed carbon content is around 10–15 wt%. Mineral content of the biomass is around 5–7 wt%. The carbon:hydrogen:oxygen molar ratio is about 1:1.6:0.6 for the samples, therefore, the chemical formula of biomass samples could be represented by C_5_H_7.8_O_3_. It is also expected that the maximum amount of H_2_ from biomass pyrolysis should be lower than the total hydrogen content of around 6.5 wt%.

**Table 1:** Properties of biomass and activated carbon.

**Characterization**		**Bamboo**	**Gumwood**	**Pine**	**Rosewood**	**Pre-heated activated carbon**
Proximate wt%	Moisture	3.7	4.8	3.6	5.6	0.4
	Volatile	74.0	76.3	77.9	72.7	0
	Fixed carbon	15.9	13.4	12.0	14.6	96.0
	Ash	6.4	5.5	6.5	7.1	3.6
Ultimate^*^ wt%	C	49.9	48.9	49.7	54.9	94.2
	H	6.5	6.5	6.6	6.6	0.8
	N	6.0	3.5	2.4	0.5	0.6
	S	0.6	0.6	0.6	0.6	0.1
	O_diff_	37.0	40.5	40.6	37.4	4.3
Intrinsic °C	T_Ig_	275	286	273	290	–
	T_P1_	321	336	307	339	–
	T_P2_	468	475	450	427	–
	T_B_	526	522	489	481	–
LHV MJ/kg		6.9	6.7	5.7	6.2	–
Lignocellulosic contents^*^ wt%	Cellulose	53.1	62.4	59.1	62.9	–
	Hemicellulose	35.0	23.2	29.7	13.0	–
	Lignin	11.9	14.4	11.2	24.1	–

* *Dry and ash free*.

Table 1 also illustrates ignition temperature (T_Ig_), peak temperature (T_P_), and burnout temperature (T_B_) of the samples. Generally, combustion profile of biomass is characterized by 2 peak temperatures, which can be attributed to the burning of cellulose & hemicellulose at 300–340°C and lignin at 420–480°C, respectively (Burhenne et al., 2013; Pang et al., 2014; Oladejo et al., 2017). The ignition and burnout temperature of biomass samples varies between 270–290° and 480–530°C, respectively and the LHV of biomass ranges between 5.7 and 6.9 MJ/Kg. These features are much lower than that of coal samples due to the higher reactivity and lower energy content of biomass samples (Omar et al., 2011; Yan et al., 2016).

Table 1 shows that cellulose content of all samples is above 50 wt% and rosewood has the highest lignin content (24 wt%). Normally, lignin is a complex 3-dimensional polymer that is more stable than cellulose and hemicellulose and its pyrolysis generates phenols and its derivatives in the bio-oil yield (Muthuraman et al., 2010; Li et al., 2011; Shi et al., 2014). Therefore, it is expected that the pyrolysis of rosewood should produce more phenols and its derivatives.

### Evaluation of Pyrolytic Yields

The distribution of products from microwave-assisted pyrolysis, conventional pyrolysis, and pyrolysis combined with activated carbon enabled reforming is listed in Table 2. During conventional pyrolysis, the yield of gaseous fraction increased with increasing temperature from 600 to 800°C. It is believed that the further cracking of pyrolytic tar at higher temperatures plays a significant role in this process (Muthuraman et al., 2010). This is further confirmed by data illustrated in Table 2 which clearly shows the reduction in the yield of bio-oil with temperature. The bio-oil yield varied from 5 to 13 wt%, whereas the gaseous fraction yield reached as high as 61.2–81.6 wt%. An increase in the gas products from 66.7 to 81.1 wt% was detected, which are associated with temperature increase from 600 to 800°C, while bio-oil yield remained lower than 11.4 wt%. It is clear that temperature significantly influences the performance of the pyrolysis process and the microwave assisted reactor generates more gaseous fractions and less bio-oil for most operating temperatures in comparison to the conventional reactor. Consequently, at 800°C, pyrolytic liquid product from MAP of gumwood, pine and rosewood was around 3.2–6.1 wt% compared to conventional reactor which ranges from 8.2 to 13.6 wt%. This indicates the higher efficiency of microwave reactors in tar cracking reactions for minimizing liquid products (Beneroso et al., 2016).

**Table 2:** Product distribution of biomass pyrolysis and reforming.

**Biomass**	**Temperature** **^**°**^C**	**Microwave-assisted pyrolysis Yields wt%**			**Conventional pyrolysis Yields wt%**		
		**Char**	**Bio-oil**	**Gaseous fraction**	**Char**	**Bio-oil**	**Gaseous fraction**
Bamboo	600	19.0	8.3	72.7	26.5	12.3	61.2
	700	17.8	7.3	74.9	17.6	9.7	72.7
	800	17.5	6.1	76.4	16.0	8.8	75.2
Gumwood	600	20.0	8.3	71.7	14.1	11.6	74.3
	700	19.0	5.2	75.8	11.4	13.5	75.1
	800	14.5	4.4	81.1	8.2	13.6	78.2
Pine	600	20.4	6.5	73.1	16.4	12.9	70.7
	700	18.1	5.2	76.7	12.9	9.6	77.5
	800	17.5	4.0	78.5	14.4	8.2	77.4
Rosewood	600	21.9	11.4	66.7	16.8	12.3	70.9
	700	22.2	6.2	71.6	12.0	9.8	78.2
	800	19.2	3.2	77.6	9.5	8.9	81.6
		**Microwave-assisted pyrolysis with reforming**			**Conventional pyrolysis with reforming**		
Bamboo	600	22.8	0.3	76.9	21.6	0.8	77.6
Gumwood	600	24.1	0.7	75.2	20.5	1.3	78.1
Pine	600	23.5	2.2	74.3	20.8	2.8	76.4
Rosewood	600	23.7	1.0	75.3	22.8	2.0	75.2

The use of SiC as a microwave receptor would result in the formation of several hot spots after attaining the reaction temperature (Li et al., 2011; Zhao H. et al., 2017). The interactions and transfer of heat energy between the biomass sample and these hot spots have substantial effect on the distribution and characteristics of the yields (Huang et al., 2016). The hot spots generated would mostly increase the yield of volatiles by enhancing the cracking of heavier volatiles and bio-oil/tar generated from the primary reaction while also gasifying the char. Simultaneously, the localized increase in temperature would also promote secondary reactions such as thermal cracking of gaseous products and polymerization of some tars to produce pyrolytic char (coke), leading to carbon deposition reactions (Fagbemi et al., 2001). Nonetheless, the formation of char in the reactor would enhance microwave absorption and consequently lead to higher temperature and faster reaction progression (Lin et al., 2014). This suggests the concurrent occurrence of several reactions in the microwave reactor and the need to optimize operating conditions to minimize such coke deposition issues while increasing gaseous yield.

During the course of microwave-assisted reaction, both the SiC and moisture contained in the biomass promptly generates heat due to their high dielectric loss tangent representing the material's property in converting absorbed microwave energy into heat (Motasemi and Afzal, 2013). When pyrolysis proceeds, volatile release in biomass begins and this results in solid char generation. Since char is also a great microwave absorber, increase in the particle's internal temperature and reactor temperature is boosted. Resultant from the volumetric heating nature of microwave, the ease of volatile release from the samples is higher than in conventional reactors, thereby generating more gas yields (Lam et al., 2017; Lo et al., 2017). However, the interaction of these gases with the SiC hot spots would result in further secondary reactions, which would also influence the overall gaseous and char yield fractions.

For rosewood, the microwave-assisted pyrolysis produced less gaseous fraction in comparison to conventional pyrolysis. This could be attributed to the high lignin content in rosewood, contributing to higher char fraction. This coupled with the higher mineral constituents of rosewood would catalytically influence reaction progression such that condensed structure formation is favored, macromolecules cracking is subdued and solid char yield increases (Shen et al., 2013). This is representative of ash inhibiting effect during microwave-assisted pyrolysis. The low gaseous yield from microwave-assisted pyrolysis of rosewood can be linked to its higher mineral contents, which is similar to the results found by other researchers in the study of burning profile of lignin (Zhao H. et al., 2017).

The distribution of products from the conventional pyrolysis and microwave pyrolysis coupled with REAC at 600°C is shown in Table 2. In comparison to the conventional and microwave pyrolysis reaction without reforming, there was an increase in gaseous product and a decrease in bio-oil yield when active carbon was adopted as reforming agent. This could mitigate potential corrosion issues experienced by conventional pyrolysis since bio-oil yield was <1 wt% in some test. Nonetheless, the reformed bio-oil fraction in conventional pyrolysis was more than that of microwave pyrolysis due to the lower initial bio-oil yield obtained with MAPB. Gaseous yield obtained from pyrolysis integrated with activated carbon enabled reforming at 600°C increased by 1.2–8.6 and 3.8–16.4 wt% for the microwave and conventional pyrolysis, respectively. This yield is similar to the gas obtained previously at 800°C without reforming. During the reforming, products distribution is affected by the release of volatiles, primary, and secondary cracking. The reduced yield of bio-oil and increased yield of gaseous products are mainly attributed to the primary cracking which aids further volatile release. The high gaseous yield can also be associated with the secondary cracking of non-condensable volatiles. When secondary cracking of non-condensable volatiles occurs, the yields of gas products and char increases because of thermal cracking. The use of activated carbon has been discovered to enhance both cracking and coking reactions of tar components via secondary reactions such as dealkylation and opening of hydroaromatic rings into gaseous fractions and polymerization reactions into char (Shamsi, 1996). It indicates that pyrolysis coupled with activated carbon enabled reforming has great potential for biomass conversion into high-value syngas at moderate operating temperatures.

### Thermal Characterization of Char

Proximate analyses of chars data are shown in Table 3. It can be seen that negligible volatile matter remained in chars, which decreased further with the increase in pyrolysis temperature such that even at such low temperature, i.e., 600°C, pyrolysis was closer to completion for microwave assisted pyrolysis in comparison to the conventionally pyrolysis. This suggests that pyrolysis temperature, reaction time, and heating rate influence the thermal decomposition of biomass and thermal properties of the resulting char. Volatile content of bio-chars obtained from microwave pyrolysis was less than that obtained from conventional pyrolysis. This is attributed to the heating mechanism of the MAPB. This unique heating process results in a mixed heat transfer mechanism during devolatilization process. Due to the low dielectric loss tangent of the biomass samples, during microwave pyrolysis, the initial release of volatiles is similar to that of conventional heating mechanisms via conduction, convection and radiation because the thermal decomposition progresses by heat transfer from SiC to biomass. This is because the SiC would effectively convert absorbed microwave into heat energy and the resultant heat exchange would dominate the reaction's temperature. Due to the temperature gradient between the SiC and biomass, the preliminary transfer of heat is external from SiC and flows to the biomass sample. This interaction between biomass and SiC will be significant and will influence the product distribution (Lin et al., 2014; Huang et al., 2016). Since the SiC is mixed evenly with biomass, the rapidity and efficiency of heat exchange between SiC and biomass would be greater due to larger surface contact between SiC and biomass in the quartz reactor than the “wall heating” mechanism of conventional heating, resulting in improved decomposition in MAPB. With the reaction progression and carbonization of biomass during the early stages of pyrolysis, the microwave absorbing properties of biomass improves with increasing dielectric loss factor (Motasemi et al., 2014). Therefore, the carbonized biomass samples can also absorb and convert microwave energy into heat via dielectric heating mechanism such that heating of the sample begins internally by energy conversion as a result of dipolar and interfacial polarization and ionic conduction mechanisms for inducing localized and instantaneous heating of the samples (Luque et al., 2012; Liew et al., 2018). This leads to further decomposition of biomass with more gaseous species generated. The fixed carbon of pyrolytic char of MAPB was higher than that from conventional pyrolysis, resulting from higher decomposition of biomass under microwave heating.

**Table 3:** Proximate analyses of pyrolytic chars^a^.

**Biomass**	**Temperature** **^°^C**	**Microwave pyrolysis**		**Conventional pyrolysis**	
		**Volatile** **wt%**	**Fixed carbon** **wt%**	**Volatile** **wt%**	**Fixed carbon** **wt%**
Bamboo	600	3.6	96.4	22.5	77.5
	700	3.2	96.8	18.1	81.9
	800	3.2	96.8	14.0	86.0
Gumwood	600	4.3	95.7	27.4	72.6
	700	4.7	95.3	15.2	84.8
	800	4.2	95.8	19.0	81.0
Pine	600	6.8	93.2	21.9	78.1
	700	6.1	93.9	18.7	81.3
	800	5.7	94.3	15.5	84.5
Rosewood	600	7.2	92.8	19.2	80.8
	700	3.8	96.2	15.5	84.5
	800	4.4	95.6	17.5	82.5

a *Moisture and ash free basis*.

Table 4 shows the intrinsic analyses of chars. The ignition, peak, and burnout temperatures of conventional pyrolytic chars are in the ranges of 360–450°, 440–520°, and 510–580°C, respectively, which are higher than the intrinsic temperatures of original biomass. This is associated with the release of most of the volatiles from the biomass during pyrolysis. Normally, for biomass, volatiles contribute significantly to the ignition. While for pyrolytic char samples, ignition is initiated by fixed carbon which is more resistant to burning and ignition. The ignition temperature, peak, and burnout temperatures of microwave chars are above 500, 600, and 700°C, respectively, which are significantly higher than those of conventional pyrolytic chars listed in Table 4. This is due to a higher degree of carbonization in the microwave reactor resulting from the combined heating mechanisms that occurs during devolatilization, leading to formation of a more graphitic char in comparison to the conventional pyrolyzer (Fan et al., 2017; Antunes et al., 2018). The proximate analysis of the char in Table 3 shows higher fixed carbon fraction in the microwave pyrolytic char than conventional pyrolytic char. This is attributed to higher extent of decomposition of biomass under microwave heating, hence lower char reactivity.

**Table 4:** Intrinsic analysis of pyrolytic char.

**Biomass**	**T_pyrolysis_** **^°^C**	**Microwave-assisted pyrolysis**			**Conventional pyrolysis**		
		**T_**Ig**_** **^**°**^C**	**T_**P**_** **^**°**^C**	**T_**B**_** **^**°**^C**	**T_**Ig**_** **^**°**^C**	**T_**P**_** **^**°**^C**	**T_**B**_** **^**°**^C**
Bamboo	600	549	664	734	368	485	560
	700	551	651	730	358	458	528
	800	547	650	715	359	466	528
Gumwood	600	549	646	716	413	512	581
	700	548	650	729	424	524	587
	800	551	645	709	417	491	559
Pine	600	550	644	723	418	444	516
	700	564	653	727	427	475	518
	800	554	650	718	447	470	534
Rosewood	600	520	648	714	368	451	541
	700	534	673	740	400	478	540
	800	521	658	728	420	484	549

### Characterization of Bio-Oil

The mass percentage of the main compounds in bio-oil was determined using semi-quantitative analysis of the chromatographic area of the detected compounds. In conventional pyrolysis at 600°C, bio-oil from biomass mainly contains phenol, catechol, benzene, xylene, furfural, and their derivatives. It is found that styrene, indene, naphthalene, and their derivatives were formed when pyrolysis at 700°C. At higher pyrolysis temperature (800°C), more biphenylene, naphthalene, phenanthrene, fluorene, anthracene, and their derivatives were formed. It is clear that the side chains of phenol or benzene derivatives were cracked at high pyrolysis temperatures. In addition to this, Polycyclic Aromatic Hydrocarbons (PAHs) were also detected in the bio-oil. styrene, a valuable chemical in industry, was produced at temperatures above 700°C. It is found that phenol, furanmethanol, cresol, catechol, and derivatives were the main compounds existing in bio-oil, which are similar to constituents of conventional pyrolytic bio-oil. However, some sulfur-containing compounds were generated in microwave pyrolytic bio-oil, such as cyclic octaatomic sulfur, dimethyl tetrasulfide or dimethyl trisulfide. Furthermore, the content of cyclic octaatomic sulfur in microwave bio-oil increased with temperatures, indicating sulfur migrates from biomass to bio-oil under microwave radiation. Similar observations about benefits of moderate microwave treatment for breaking the C–S bonds has been discovered by other studies (Tao et al., 2016). In addition, D-allose is another typical compound in microwave pyrolytic bio-oil, which is absent in conventional pyrolytic bio-oil. In comparison with CPB, the concentrations of phenol, phenolics, furfural, and benzofuran in MAPB pyrolytic bio-oil decreased. This suggests that microwave irradiation enhances the cracking of furfural and benzofuran.

When microwave pyrolysis is coupled with reforming, the reduced quantity of bio-oil is explained by further cracking of bio-oil compounds in the activated carbon-enabled reforming zone. This also results in variation in composition such that cyclic octaatomic sulfur and phenol became the main constituents, while PAHs became minor. In comparison to the composition of MAPB bio-oil at 600°C, it is clear that the MAPB-REAC derived bio-oil has experienced additional breakdown of allose, -methoxy and –dimethoxy groups during reforming. In addition, the compounds of bio-oil produced from MAPB-REAC and CPB-REAC had fewer methoxy groups. This suggests that activated carbon would be an important asset in microwave reforming for producing lower bio-oil yields while narrowing product distribution significantly. A non-oxidized atmosphere is created by the active carbon which improves the cracking of oxygenated volatiles into gaseous molecules rather than generating tar (Fernández et al., 2009). The results from this study demonstrates that activated carbon reduced the yield of bio-oil but enhanced the yield of gaseous product. In contrast, it was reported that the addition of activated carbon showed significant increase in both bio-oil and gas yields during the microwave-assisted pyrolysis of corn stover (Salema and Ani, 2011). However, the difference can be attributed to their use of char as the microwave absorber rather than the reforming agent.

### Gaseous Fraction Characterization

The gaseous yield composition of the samples at different temperatures is shown in Table 5. The result reveals syngas composition of 53–81 vol% with CO content of ≤ 57 vol% and H_2_ content of ≤ 25 vol%. Similarly, the CO_2_ and CH_4_ content were within the ranges of 7–24 vol% and 13–17 vol%, respectively. This was slightly different for rosewood whose pyrolytic gas consisted of lower H_2_ (0.8–20 vol%) and CO_2_ (3–9 vol%) fractions, and higher CO (49–67 vol%) and CH_4_ (15–22 vol%) contents. The syngas fraction was discovered to increase with increasing temperature from 600 to 800°C and the C2–C4 hydrocarbon gases were found in minor quantities. The release of volatiles from biomass, thermal cracking of bio-oil at higher temperatures and reactions between various pyrolytic compounds consequently results in the gaseous product formation (Bridgwater, 2012). Composition of the pyrolytic gas products changed with increasing temperature such that there is a discernible increase in H_2_ and syngas (CO+H_2_) and decrease in CO_2_ concentration. In addition, there was an increase in the H_2_:CO ratio with increasing temperature resulting from H_2_ increase (Bridgwater, 2012).

**Table 5:** Compositions of the gaseous fraction.

**Biomass**	**Temperature** **^**°**^C**	**Microwave-assisted pyrolysis vol%**						**Conventional pyrolysis vol%**					
		**CO_**2**_**	**CH_**4**_**	**CO**	**H_**2**_**	**Syngas**	**H_**2**_/CO**	**CO_**2**_**	**CH_**4**_**	**CO**	**H_**2**_**	**Syngas**	**H_**2**_/CO**
Bamboo	600	23.4	1.0	44.3	29.6	73.9	0.67	24.2	15.7	44.0	11.7	55.7	0.27
	700	16.8	4.6	34.3	43.3	77.6	1.26	18.0	16.0	45.3	15.1	60.4	0.33
	800	14.3	6.4	29.1	48.2	77.3	1.66	13.9	16.7	42.3	21.4	63.6	0.51
Gumwood	600	13.0	5.5	41.5	38.4	79.9	0.93	15.4	14.6	53.1	11.6	64.7	0.22
	700	14.7	4.6	37.7	41.8	79.4	1.11	7.2	17.0	57.0	13.4	70.5	0.24
	800	10.0	5.6	35.1	47.6	82.7	1.36	9.2	14.9	49.4	21.3	70.7	0.43
Pine	600	14.2	4.0	38.2	42.4	80.6	1.11	19.8	13.4	47.2	15.3	62.5	0.32
	700	14.0	3.8	38.0	43.2	81.2	1.14	13.2	15.5	49.4	16.9	66.3	0.34
	800	12.9	5.9	33.4	46.0	79.4	1.38	12.6	15.7	45.2	21.2	66.4	0.47
Rosewood	600	21.4	8.7	36.2	32.6	68.9	0.90	3.2	22.5	67.1	0.8	67.9	0.01
	700	10.3	4.0	41.7	43.0	84.7	1.03	3.8	21.2	62.7	5.2	67.9	0.08
	800	8.6	5.6	38.0	46.3	84.3	1.22	9.5	15.3	49.3	20.0	69.4	0.41
		**Microwave-assisted pyrolysis and reforming vol%**						**Conventional pyrolysis and reforming vol%**					
Bamboo	600	11.2	2.9	29.4	55.7	85.1	1.89	20.5	15.2	40.7	21.4	62.1	0.53
Gumwood	600	15.8	4.1	35.0	44.1	79.1	1.26	20.2	10.4	44.0	23.0	67.0	0.52
Pine	600	16.2	3.4	36.0	43.8	79.8	1.22	24.5	10.8	38.6	23.8	62.4	0.62
Rosewood	600	16.9	3.7	33.7	44.7	78.4	1.32	18.6	10.3	39.7	29.4	69.1	0.74

*Difference to 100% for listed gaseous fraction (CO_2_ + CH_4_ + CO + H_2_) is attributed to C_2_-C_4_ hydrocarbons which makes up ≤ 2 vol% for microwave assisted pyrolysis and ≤ 7.1 vol% for conventional pyrolysis*.

Although the gaseous fraction from MAPB is similar to that of CPB as presented in Table 2, the H_2_ selectivity under microwave irradiation was higher, particularly at higher temperatures as seen in Table 5, which shows the composition of the microwave pyrolytic gaseous fraction. The fraction of CO_2_ in the gas yield from the pyrolysis of most biomass samples decreased with increasing temperature. The methane fraction in the MAPB derived gas product were <6 vol% which is quite lower than that from CPB. It is generally believed (Borges et al., 2014a; Lam et al., 2016b) that both dry reforming of methane and thermal cracking of methane can be enhanced by microwave heating which increases the syngas fraction of the gaseous yield. In addition, the dehydrogenation reaction (aromatization, condensation, and alkene formation reactions) of char and oil, as well as dehydrogenation of ethane to ethylene would also contribute to the increase in hydrogen gas yield.

In the MAPB derived gas, the fraction of CO was reduced in comparison to that in conventional pyrolysis and this is mainly resulting from the influence of microwave heating. During the CPB reaction, pyrolytic char begins formation when reactor temperature is within the range of 150–300°C as a result of the breaking of linkages in alkyl chains (Domínguez et al., 2007; Yu et al., 2009; Ferrera-Lorenzo et al., 2014), depolymerizing of cellulose and xylan of biomass (Fidalgo et al., 2010). When the temperature is in the range of 150–300°C, conventional pyrolysis and microwave-assisted pyrolysis showed similar behavior with negligible pyrolysis progression (heat transfer in MAPB was dominated by conduction and convection). With further increase in temperature, microwave-assisted pyrolysis process was accelerated because of the high microwave absorbing capacity of the bio-char generated which contributes to microwave radiation transformation into heat energy (Gadkari et al., 2016; Li et al., 2016). In conventional pyrolysis, the generation of CO has been linked to the breakage of alkyl aryl linkages, the disintegration of acetyl, -COOH, –OCH_3_, and other secondary reactions that occurs between 200 and 400°C. At higher temperatures above 400°C, CO is generated from alkyl chain transformations and the chemical alterations in short substituents of aromatic rings (Collard and Blin, 2014). In contrast, higher temperatures during MAPB enhances thermal cracking; dry reforming and dehydrogenation reactions of the char, oil and gases, and this consequently results in an increase in light condensable bio-oil and incondensable gases. This results in higher fractions of CH_4_ and H_2_ rather than CO.

Even with the decrease in the proportion of CO in the gaseous yield of MAPB, the total syngas fraction was ≥10 vol% higher than in CPB under similar operating conditions. Higher H_2_ fraction was obtained from MAPB with the highest syngas yields of ≈85 vol%. As the reaction temperature increased from 600 to 800°C, the produced H_2_ increased under MAPB demonstrating that microwave irradiation boosts the yield of H_2_ via enhanced tar cracking, devolatilization process and heterogeneous reactions between gaseous species and steam/CO_2_ at higher temperatures. Nevertheless, there was a reduction in CH_4_ fraction obtained from the microwave heating compared to the conventional heating. This is attributed to the enhancement of CH_4_ thermal cracking reaction on char surface as a result of microwave-induced local high temperature sites (hot spots) (Yang et al., 2007). This would result in pyrolytic coke yield and hydrogen gas. As a result, the percentage of CH_4_ decreases while the content of H_2_ and solid coke increases. Contrarily, thermal cracking of CH_4_ does not occur during conventional heating at a temperature <800°C (Menéndez et al., 2010; Widyawati et al., 2011). In terms of CH_4_, conventional pyrolysis is superior to microwave-assisted pyrolysis.

**Figure 2:**
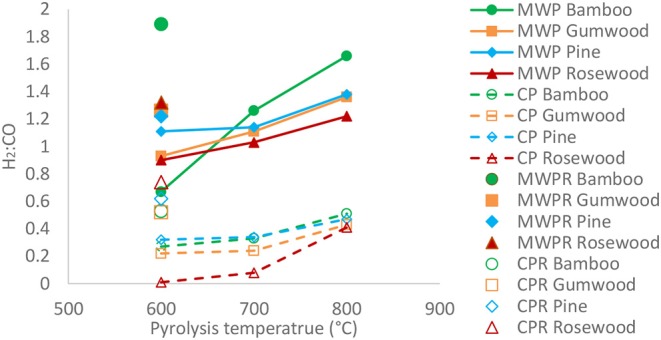
Effects of pyrolysis temperature on H_2_:CO in gaseous fraction.

Overall, the gaseous fraction derived from MAPB contained significantly higher proportions of syngas (CO + H_2_) in comparison to the syngas yield from conventional pyrolysis under similar conditions (Fidalgo et al., 2008b). An additional benefit of MAPB is the potential of extracting higher gaseous and bio-oil yields at low temperatures compared to CPB of similar temperature (Fidalgo et al., 2008a,b, 2010). This can be attributed to the unique features of microwave heating. In Figure 2, it is clear that the H_2_/CO mole ratio increases with temperature, which is significantly higher for MAPB than CPB. The syngas with higher H_2_/CO is synonymous to higher calorific value and can be utilized directly in gas engines or further processed for chemicals or liquid fuels. The highest H_2_/CO ratio was 1.66, which was obtained in the MAPB of bamboo at 800°C.

### Gaseous Yield Characterization After Reforming

The use of a reforming agent has considerable impact on the product distribution and their compositions. The influence of reforming can be seen in Table 2 which lists the constituents of pyrolytic gas after the activated carbon-enabled reforming process. In comparison to CPB, an increase in the CO_2_ and H_2_ fraction was detected after reforming while CO and CH_4_ fraction reduced. This is associated with the improved thermal cracking of CH_4_ and other light hydrocarbons, and enhanced C+CO_2_ reactions due to the presence of activated carbon, which consequently increases CO_2_ and H_2_ generation via enhanced water gas shift reaction. Reforming coupled with CPB resulted in a percentage of syngas of 69.1 vol% and a H_2_ fraction of ~30 vol%.

Similarly, higher H_2_ selectivity was observed in MAPB coupled with reforming, such that the H_2_ content was as high as 55 vol% for bamboo. In comparison to conventional pyrolysis of bamboo coupled with reforming, a 35% increase in H_2_ yield was obtained from the microwave-enhanced reforming. This results in a higher syngas fraction and an increase in H_2_/CO ratio.

### Evaluation of Energy Conversion

LHV of the biomass samples is presented in Table 1, which varies from 5.7 to 6.9 MJ/Kg. The LHV of gaseous fraction were calculated based on combustible gas components like CH_4_, CO and H_2_. For gas products, LHV was found to be in the range of 15.6–24.6 MJ/kg. From Table 6, it is evident that the LHV of gaseous fraction increased with pyrolysis temperature, which is associated with the high yield of syngas.

**Table 6:** Energy distribution in various pyrolytic products.

**Biomass**	**Temperature** **^**°**^C**	**Microwave-assisted pyrolysis LHV MJ/kg**			**Conventional pyrolysis LHV MJ/kg**		
		**Char**	**Bio-oil**	**Gaseous fraction**	**Char**	**Bio-oil**	**Gaseous fraction**
Bamboo	600	13.8	6.0	15.6	16.6	5.0	18.5
	700	13.1	4.6	21.3	15.7	4.7	18.8
	800	13.1	6.1	24.6	16.3	5.5	20.4
Gumwood	600	14.4	5.7	19.2	15.1	5.7	16.9
	700	12.9	4.8	20.1	15.1	3.9	17.4
	800	11.9	5.3	22.2	14.7	5.4	18.7
Pine	600	14.4	6.0	19.9	14.4	5.2	17.7
	700	13.4	5.1	20.0	14.5	5.5	18.3
	800	12.0	6.2	22.5	15.1	5.6	19.6
Rosewood	600	16.6	4.6	20.6	17.2	6.3	16.6
	700	13.7	7.1	19.3	16.8	5.2	17.1
	800	13.3	6.2	21.2	15.9	5.9	18.6
		**Microwave-assisted pyrolysis with reforming**			**Conventional pyrolysis with reforming**		
Bamboo	600	14.1	6.8	24.5	17.6	7.4	20.3
Gumwood	600	14.0	6.6	21.0	14.2	5.6	18.2
Pine	600	13.1	8.5	20.4	12.9	5.8	19.5
Rosewood	600	15.1	7.5	21.3	14.3	6.3	19.9

Microwave char has a lower LHV than that of chars derived from conventional pyrolysis (14 to 17 MJ/kg). Low LHV could also be attributed to the higher extent of decomposition of biomass under microwave heating, leading to the chars containing less carbon. LHV of the microwave pyrolytic gas products was around 15–26 MJ/kg. This is much higher than that of gaseous fraction derived from conventional pyrolysis, as listed in Table 6. It is evident that gaseous fraction via MAPB was more suitable to be applied as a fuel or feed for chemical production.

**Figure 3:**
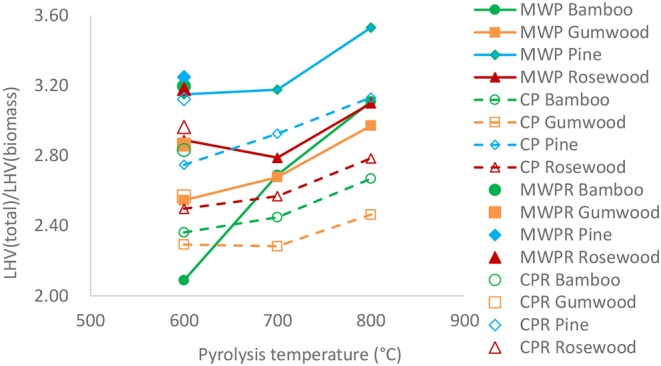
Ratio of LHV (pyrolysis products) to LHV (biomass).

LHV of pyrolytic products and its ratio to LHV of biomass were calculated and illustrated in Figure 3, which shows that the ratio of LHV (products) to LHV (biomass) increased with temperature. Therefore, high pyrolysis temperature favors higher degree of energy conversion from biomass into pyrolytic products. When temperature rised from 600 to 800°C, LHV of total pyrolytic products also increased. The ratio of LHV (products) to LHV (biomass) is in the range between 2.1 and 3.5, which means the internal energy of total pyrolytic products is higher than that of feedstock due to energy transfer mechanism from the microwave and electric heating. The ratio for microwave-assisted pyrolysis was higher than that for conventional pyrolysis at the same pyrolysis temperature, which means that under the same temperature, microwave heating has the potential to increase the internal energy of the products more than conventional pyrolysis. Chemical energy in biomass and electromagnetic energy of microwave irradiation are converted into energy of pyrolytic products. Since both MAPB and CPB were carried out under similar conditions, such as reactor size, sample loading weight, pyrolysis temperature and reaction time, it can be concluded that the energy from products of microwave-assisted pyrolysis is much higher than that of conventional pyrolysis.

LHVs of products derived via different pyrolysis methods are also illustrated in Table 6 and Figure 3. The total LHV of the products produced via MAPB-REAC is slightly higher than that of CPB-REAC, except for rosewood, whose mineral contents were higher than those of any other biomass. In Figure 3, compared with MAPB only, the reforming process also increased the LHV (products)/LHV (biomass), which can be attributed to the enhancement associated with microwave irradiation. In Table 6, the LHVs of the pyrolytic gas products from the reforming process was around 17–20 MJ/kg for conventional pyrolysis and 17–25 MJ/kg for microwave-assisted pyrolysis, respectively. The LHV of gaseous fraction produced by MAPB-REAC was higher than that of conventional method and is more suitable to be applied as fuel or feed gas for the synthesis of chemicals. Additionally, Table 7 depicts the energy recovery efficiency of the microwave assisted pyrolysis of biomass with an efficiency of 5.56–6.91%, 6.67–7.16%, and 7.41–8.25% for reactions done at 600, 700, and 800°C. Increase in efficiency was observed when the reforming stage was incorporated such that an increase from 5.56 to 6.91% of MAPB at 600°C to 7.12–8.49% with MAPB-REAC signifying reaction enhancement.

**Table 7:** Energy recovery efficiency of the microwave assisted pyrolysis.

**Biomass**	**T/^**°**^C**	**Microwave-assisted pyrolysis, MJ per 5 g used for run**								
		**Char**	**Bio-oil**	**Gaseous fraction**	**Total output energy**	^*^Microwave Energy (MJ/Kg)	^**^Electrical energy (MJ)	**Biomass energy (MJ)**	**Total input energy (MJ)**	**Energy conversion efficiency (%)**
Bamboo	600	0.013	0.002	0.057	0.072	1.013	1.266	0.035	1.300	5.56
	700	0.012	0.002	0.080	0.093	1.013	1.266	0.035	1.300	7.16
	800	0.011	0.002	0.094	0.107	1.013	1.266	0.035	1.300	8.25
Gumwood	600	0.014	0.002	0.069	0.086	1.013	1.266	0.034	1.299	6.59
	700	0.012	0.001	0.076	0.090	1.013	1.266	0.034	1.299	6.90
	800	0.009	0.001	0.090	0.100	1.013	1.266	0.034	1.299	7.68
Pine	600	0.015	0.002	0.073	0.089	1.013	1.266	0.029	1.294	6.91
	700	0.012	0.001	0.077	0.090	1.013	1.266	0.029	1.294	6.97
	800	0.011	0.001	0.088	0.100	1.013	1.266	0.029	1.294	7.73
Rosewood	600	0.018	0.003	0.069	0.090	1.013	1.266	0.031	1.297	6.90
	700	0.015	0.002	0.069	0.087	1.013	1.266	0.031	1.297	6.67
	800	0.013	0.001	0.082	0.096	1.013	1.266	0.031	1.297	7.41
		**Microwave-assisted pyrolysis with reforming**								
Bamboo	600	0.016	0.000	0.094	0.110	1.013	1.266	0.035	1.300	8.49
Gumwood	600	0.017	0.000	0.079	0.096	1.013	1.266	0.034	1.299	7.39
Pine	600	0.015	0.001	0.076	0.092	1.013	1.266	0.029	1.294	7.12
Rosewood	600	0.018	0.000	0.080	0.098	1.013	1.266	0.031	1.297	7.59

* *Microwave operation assumed at 50% of maximum power (3 KW) for 75% of reaction time (15 min) due to on/off operation to maintain temperature*.

** *Electrical energy to Microwave generation efficiency of ~80%*.

### Mechanism of Microwave-Assisted Reforming

Normally carbon materials, for instance char and activated carbon, can be applied as catalysts for heterogeneous reactions under microwave irradiation (Dufour et al., 2009; Lam et al., 2017). The dielectric loss tangent [tanδ, the ratio of dielectric loss factor (ε”) to dielectric constant (ε')] of active carbon at an operating frequency of 2.4GHz is between 0.57 and 0.8 (Fernández et al., 2009; Luque et al., 2012). This is higher in comparison to the tanδ of SiC (0.25) (Idem et al., 1996) and biomass(<0.1) (Li et al., 2016). This makes activated carbon a better microwave absorber.

Remarkably, activated carbon actively participates in the reforming process as a reactant to generate a reductive atmosphere which boosted the breakdown of oxygenated compounds in the volatiles. This was further validated by the lack of methoxy and -dimethoxy groups in the bio-oil obtained from MAPB which indicates that redox reaction took place between the bio-oil and activated carbon. With the help of activated carbon reforming, oxygen migrates from condensable bio-oil to incondensable gas fraction which results high pyrolytic gas yield and low bio-oil yield. Similarly, this was also made evident by the reaction (1) which promotes CO formation due to activated carbon reaction with CO_2_ in MAPB. Analogous observations were detected between CO_2_ and char in sewage sludge pyrolysis using a microwave reactor (Menéndez et al., 2010). In addition, the activation of the surface of the carbon particles by the microwave radiation also catalyzes and enhances the reaction (2) (Atwater and Wheeler, 2004). Consequently, during the reforming reaction with MAPB, concurrent reaction of oxygen-containing hydrocarbons, CO_2_ and steam with activated carbon can occur.

(1) $$\begin{array}{l} \text{C}\left(\text{s}\right)+{\text{CO}}_{2}\left(\text{g}\right)\to 2\text{CO}\left(\text{g}\right) \end{array}$$

(2) $$\begin{array}{l} \text{C}\left(\text{s}\right)+{\text{H}}_{2}\text{O}\left(\text{g}\right)\to \text{CO}\left(\text{g}\right)+{\text{H}}_{2}\left(\text{g}\right) \end{array}$$

Furthermore, It has been established that microwave heating promotes various catalytic reactions (Motasemi et al., 2014). Char also enhances this as well via the heterogeneous decomposition of main organic gases at comparatively lower temperatures (<500°C) (Domínguez et al., 2008; Motasemi et al., 2014) and the promotion of gas phase ancillary cracking (Fidalgo et al., 2008b). This indicates the role of activated carbon as a catalyst in volatile cracking and this catalytic activity is further enhanced by microwave irradiation (Liew et al., 2018). This indicates the multiple functions of activated carbon as a microwave absorber, a catalyst and a reactant. From Table 2, it is clear that more gaseous fraction and less bio-oil (1 and 5 wt%) were produced when activated carbon was used in both microwave and conventional pyrolysis coupled with reforming.

## Conclusions

To summarize, it is found that microwave-assisted pyrolysis coupled with activated carbon enabled reforming resulted in an enhanced yield of gaseous product, higher H_2_ selectivity and reduced yield of bio-oil. The highest bio-oil yield was lower than 3 wt%, and the highest gaseous fraction was around 76 wt%, which contains 85 vol% of syngas and 46.7 vol% of H_2_. The thermal cracking and reforming reactions were improved by microwave irradiation, resulting in the production of a H_2_-rich syngas. The activated carbon enabled reforming process favors dehydration and deoxidation reactions by the creation of a non-oxidizing atmosphere, which further promotes hydrogen production. The findings of this study demonstrate that the coupling of activated carbon enabled reforming with microwave-assisted pyrolysis favors the conversion of biomass into H_2_-rich gas and raises energy conversion efficiency.

## Data Availability Statement

All datasets generated for this study are included in the article/supplementary material.

## Author Contributions

All authors listed have made a substantial, direct and intellectual contribution to the work, and approved it for publication.

### Conflict of Interest

The authors declare that the research was conducted in the absence of any commercial or financial relationships that could be construed as a potential conflict of interest.
